# Treatments for dry age-related macular degeneration: therapeutic avenues, clinical trials and future directions

**DOI:** 10.1136/bjophthalmol-2020-318452

**Published:** 2021-03-19

**Authors:** Thales Antonio Cabral de Guimaraes, Malena Daich Varela, Michalis Georgiou, Michel Michaelides

**Affiliations:** 1 Institute of Ophthalmology, University College London, London, London, UK; 2 Moorfields Eye Hospital NHS Foundation Trust, London, London, UK

**Keywords:** genetics, angiogenesis, degeneration, retina, macula

## Abstract

Age-related macular degeneration (AMD) is the leading cause of irreversible blindness in the developed world. The identification of the central role of vascular endothelial growth factor (VEGF) in the pathogenesis of neovascular AMD and the introduction of anti-VEGF agents as gold-standard treatment, have drastically changed its prognosis—something yet to be seen in dry AMD. Several therapeutic avenues with a wide variability of targets are currently being investigated in dry AMD. The approaches being investigated to reduce the rate of disease progression include, (1) drugs with antioxidative properties, (2) inhibitors of the complement cascade, (3) neuroprotective agents, (4) visual cycle inhibitors, (5) gene therapy and (6) cell-based therapies. A number of early phase clinical trials have provided promising results, with many more ongoing and anticipated in the near future. In this review, we aim to provide an update of the interventional trials to date and future prospects for the treatment of dry AMD.

## Introduction

Age-related macular degeneration (AMD) is a multifactorial condition and the leading cause of irreversible blindness in the elderly population, with the number of affected patients predicted to be 288 million by 2040.^1 2^ Its progressive and irreversible nature translates to a significant societal cost burden and increased health resource utilisation.^3–5^ Moreover, ageing is the main risk factor to develop AMD, hence, as life expectancy increases, this burden will ultimately become greater in the foreseeable future.

There are several classifications of AMD, based on clinical and imaging findings.^6–8^ One published approach suggests that early and intermediate stages are characterised by variable size and amount of drusen and the presence of pigmentary abnormalities, and the late stage displays signs of advanced disease, such as geographic atrophy (GA) and neovascularisation.^9^ AMD can be pragmatically divided into two groups according to the presence or absence of neovascularisation: (1) dry or non-neovascular AMD (or GA) and (2) wet or neovascular AMD (nAMD). The implication of vascular endothelial growth factor (VEGF) in the pathogenesis of nAMD and the introduction of anti-VEGF as the gold-standard treatment has drastically changed its prognosis—something yet to be seen in dry AMD.

Several therapeutic approaches have been investigated for dry AMD and are currently in different stages of clinical trial, including antioxidative therapy, drug treatments targeting multiple pathways, cell and gene directed therapies, as well as retinal implants. This article aims to provide an update on prioritised current therapeutic avenues for dry AMD and future treatment prospects.

## Pathogenesis of dry AMD

AMD is a complex multifactorial disease whose pathogenesis is incompletely understood and evolving, with a detailed evaluation behind the scope of this review. Several factors are believed to contribute to its pathogenesis, including genetic,^10^ oxidative stress,^11^ environmental,^12–14^ inflammatory^15^ and ischaemic.^16^ The presence of drusen is considered the hallmark of earlier stages of AMD, which may enlarge, become confluent and evolve to drusenoid retinal pigmented epithelium (RPE) detachments.^17^ This causes a disruption in the interaction between Bruch’s membrane and RPE, inhibiting RPE function, including the crucial ability to transport photoreceptor debris across Bruch’s membrane to the choriocapillaris, further facilitaing accumulation of lipofuscin and other related products including A2E.^18^

Drusen contain several proinflammatory factors including complement pathway components (and lipofuscin-related products), which have been identified as one of the main contributors to AMD development.^19^ Several studies suggest that complement pathway activation and the consequent membrane attack complex (MAC) play a key role in choriocapillaris loss and in the development of AMD and GA.^20–22^ Mullins *et al* correlated vascular loss and choriocapillaris density with the extent of drusen, suggesting that in early stages, the deposition of complement complexes induces loss of choriocapillaris and drusen formation.^23^ Subsequently, the same group observed the deposition of MAC in the outer part of Bruch’s membrane prior to choriocapillaris loss and identified that the high-risk allele Y402H in *CFH* (OMIM #134370) is associated with elevated levels of MAC and increased risk of choriocapillaris loss in early AMD disease.^24^ Polymorphisms in multiple other genes have also been associated with increased risk of developing AMD, including genes involved in the complement cascade, *ARMS2* (OMIM #611313), *ABCA4* (OMIM #601691), and *HTRA1* (OMIM #602194).^25 26^ It is still unclear if these genetic risk alleles are associated with the development of either wet or dry AMD, or both.

## Treatments for dry AMD

Currently, the management of dry AMD consists of observation, regular follow-up evaluations and documentation, for timely recognition of visual function deterioration with appropriate rehabilitation and early choroidal neovascularisation (CNV) detection. Several therapeutic avenues are being investigated aiming at: (1) disease prevention, (2) halting disease progression and/or (3) restoration of vision. In this section, we summarise the most promising pathways of treatment and the ongoing clinical trials (table 1).

**Table 1:** Summary of ongoing clinical trials targeting dry AMD

Drug category/name	Clinical trial ID (NCT #)	Study phase	Route of delivery	Status	Sponsor	Location	Number of patients
**Antioxidative**							
AREDS	NCT00000145	Phase III	Oral	Completed	National Eye Institute (NEI)	USA	3640
AREDS2	NCT00345176	Phase III	Oral	Completed	National Eye Institute (NEI)	USA	4203
OT-551	NCT00306488	Phase II	Topical	Completed	National Institutes of Health Clinical Center (CC)	USA	11
**Reduction of toxic bryproducts**							
GSK933776	NCT01342926	Phase II	IV	Completed	GlaxoSmithKline	USA	191
RN6G	NCT01577381	Phase II	IV	Terminated	Pfizer	USA	10
**Visual cycle modulators**							
ACU-4429	NCT01802866	Phase IIb/III	Oral	Completed	Kutoba Vision Inc	USA	508
Fenretinide	NCT00429936	Phase II	Oral	Completed	Sirion Therapeutics, Inc	USA	246
C20-D3-vitamin A (ALK-001)	NCT03845582	Phase III	Oral	Ongoing—recruiting	Alkeus Pharmaceuticals, Inc	USA	300
**Anti-inflammatory and complement inhibition**							
Eculizumab	NCT00935883	Phase II	IV	Completed	Philip J. Rosenfeld, MD	USA	30
Lampalizumab	NCT02247531	Phase III	Intravitreal	Terminated	Hoffman-La Roche	Multicenter	906
Lampalizumab	NCT02247479	Phase III	Intravitreal	Terminated	Hoffman-La Roche	Multicenter	975
Sirolimus (rapamycin)	NCT00766649	Phase I/II	Subconjunctival	Completed	National Eye Institute (NEI)	USA	11
Avacincaptad pegol (Zimura)	NCT02686658	Phase II/III	Intravitreal	Completed	IVERIC bio, Inc.	Multicenter	286
Pegcetacoplan (APL-2)	NCT02503332	Phase II	Intravitreal	Completed	Apellis Pharmaceuticals Inc.	Multicenter	246
Pegcetacoplan (APL-2)	NCT03525600	Phase III	Intravitreal	Ongoing—not recruiting	Apellis Pharmaceuticals Inc.	Multicenter	600
Pegcetacoplan (APL-2)	NCT03525613	Phase III	Intravitreal	Ongoing—not recruiting	Apellis Pharmaceuticals Inc.	Multicenter	600
Tedisolumab (LFG316)	NCT01527500	Phase II	Intravitreal	Completed	Novartis Pharmaceuticals	USA	150
Risuteganib	NCT03626636	Phase II	Intravitreal	Completed	Allegro Ophthalmics	USA	42
**Neuroprotection**							
Ciliary nerve trophic factor	NCT00063765	Phase I	Intravitreal	Completed	National Eye Institute (NEI)	USA	10
Ciliary nerve trophic factor	NCT00447954	Phase II	Intravitreal	Completed	Neurotech Pharmaceuticals	USA	51
Brimonidine tartrate	NCT00658619	Phase II	Intravitreal	Completed	Allergan	Multicenter	113
Brimonidine tartrate	NCT02087085	Phase IIb	Intravitreal	Terminated	Allergan	Multicenter	303
**Gene therapy**							
AAVCAGsCD59	NCT03144999	Phase I	Intravitreal	Completed	Hemera Biosciences	USA	17
GT005	NCT03846193	Phase I/II	Subretinal	Ongoing - Recruiting	Gyroscope Therapeutics	UK	35
**Cell-based therapies**							
Palucorcel (CNTO-2476)	NCT01226628	Phase I/II	Subretinal	Completed	Janssen Research & Development, LLC	USA	35
MA09-hRPE	NCT01344993	Phase I/II	Subretinal	Completed	Astelas Institute for Regenerative Medicine	USA	9
CPCB-RPE1	NCT02590692	Phase I/IIa	Subretinal	Ongoing—not recruiting	Regenerative Patch Technologies	USA	16
**Mitochondrial enhancers**							
Elamipretide	NCT02848313	Phase I	Subcutaneous	Completed	Stealth Biotechnologies Inc	USA	40
Elamipretide	NCT03891875	Phase II	Subcutaneous	Ongoing—recruiting	Stealth Biotechnologies Inc	USA	180
**Nanosecond laser therapy**							
2RT nanosecond laser	NCT01790802	Not applicable	Retinal active laser therapy	Completed	Center for Eye Research Australia	Australia	292

AMD, age-related macular degeneration.

### Nutritional supplementation and antioxidant therapy

Oxidative damage from various sources, such as smoking, UV light exposure and oxidative stress to the retina have been strongly linked with AMD. Hence, treatments that reduce the accumulation of reactive oxygen species may be a potential therapeutic intervention.

#### Age-related eye disease study (AREDS) supplements

AREDS was a double-masked, randomised and multicentre trial (n=3640; NCT00000145) that was designed to determine the protective effects of antioxidant supplementation in patients with AMD. The participants were divided into four groups based on disease severity. The results showed that a daily dose of vitamin E (400 IU), C (500 mg), Beta-carotene (15 mg), cupric acid (2 mg) and zinc oxide (80 mg) reduced the odds of developing advanced AMD in up to 34% of the subjects with high-risk characteristics (groups 3 and 4). Moreover, follow-up of these participants over a 12-year period demonstrated that subjects who had the highest omega-3 fatty acids intake were 30% less likely to develop central GA and nAMD.^27^

Subsequently, in 2006, a new placebo-controlled phase III AREDS study (AREDS2; n=4203; NCT00345176) commenced to determine whether adding lutein + zeaxanthin, omega-3 long-chain polyunsaturated fatty acids (docohexaenoic acid (DHA) and eicosapentaenoic acid (EPA)), or a combination of both to the AREDS formulation would decrease the risk of developing advanced AMD. Addition of lutein + zeaxanthin, DHA + EPA, or both, to the AREDS formulation in primary analyses did not further reduce risk of progression to advanced AMD. More lung cancers, mostly in former smokers, were noted in the beta-carotene group (n=23), when compared with the group without (n=11). This finding led to the substitution of the carotenoid for lutein + zeaxanthin in the AREDS formula.^28 29^ In 2017, a pooled systematic meta-analysis concluded that, although some patients with higher risk of progression to more advanced stages of AMD may experience delay in progression with antioxidant vitamin and mineral supplementation, lutein and zeaxanthin-containing supplements may have little to no effect on the disease progression.^30^

#### 1-hydroxy-4-cyclopropanecarbonyloxy-2,2,6,6-tetramethylpiperidine hydrochloride (OT-551)

OT-551 is a molecule with anti-inflammatory and antioxidant effects that was found to protect against light-induced degeneration in the RPE of rats.^31^ An open-label phase II trial (n=11; NCT00306488) administered topical 0.45% OT-551 in a randomly assigned eye of participants with bilateral GA. No serious adverse events were noted in the 11 participants. The mean change in best-corrected visual acuity (BCVA) at 2 years was +0.2 ± 13.3 letters in the study eye versus −11.3 ± 7.6 letters in the non-study eye. No statistical significant differences were found between the study and contralateral eye in microperimetry measurements, contrast sensitivity, area of GA and total drusen area from baseline, with benefits deemed as limited or without benefit, in the delivered mode and concentration.^32^

### Reduction of toxic byproducts

#### β-amyloid

Another pathway being explored is targeting β-amyloid, a major endogenous protein underlying the aetiology of, for example, Alzheimer’s disease.^33^ It has been identified as a component of drusen and it is a known activator of the complement cascade. In a placebo-controlled phase II study (n=191; NCT01342926) conducted in patients with GA secondary to AMD, intravenous transfusion of GSK933776 (an anti-amyloid β monoclonal antibody) was deemed well-tolerated and safe. However, there was no clinically meaningful improvement in visual function or decrease in the rate of GA enlargement.^34^ Similarly, RN6G, another antiamyloid β monoclonal antibody has been evaluated in a phase II placebo-controlled trial (n=10; NCT01577381), administered intravenous in patients with GA. The study was stopped early by the sponsor, and not enough subjects/data were recruited/collected for meaningful analyses.^35^

GAL-101 (formerly known as MRZ-99030) is a dipeptide that has been shown to prevent the formation of oligomeric β-amyloid species.^36^ In a phase I trial (n=70; NCT01714960), GAL-101 was administered as an eyedrop in subjects with glaucoma. The drug was deemed safe, with a low frequency of side effects.^37^ A phase II trial is in development to determine its safety and efficacy in both glaucoma and dry AMD.^38^

### Visual cycle modulators

Modulation of the visual cycle by targeting participating enzymes, delivery of vitamin A to the cycle or removal of associated toxic waste products may be beneficial.

#### Emixustat hydrochloride

Oral administration of ACU-4429 (emixustat hydrochloride) has been investigated in the SEATTLE study (n=508; NCT01802866), a multicentre, randomised, phase IIb/III trial. The primary efficacy endpoint was the mean annual growth rate of total GA area, as measured by fundus autofluorescence (FAF). Although the drug was found to be safe, on average, GA lesions in the treated group progressed at a similar rate to the placebo group.^39^

#### Fenretinide

Fenretinide is a synthetic derivative of vitamin A that binds to the serum retinol-binding protein (RBP), allowing a rapid elimination of fenretinide–RBP complex through urine. In animal models, it was found to reduce circulating RBP–retinol complex levels in a dose-dependent manner and lower the production of A2E in abca4 ^−/−^ mice.^40^ A phase II, placebo-controlled, proof of concept trial, studied the efficacy of oral fenretinide in patients with GA due to AMD (n=246; NCT00429936). The drug was found to be safe but associated with side effects including significantly delayed dark adaptation. However, the achieved reduction of GA growth rate in treated patients (both high and low dose) was not statistically significant, compared with the placebo group.^41^

#### Deuterated vitamin A

A modified form of vitamin A, with a deuterium isotope replacement at carbon 20 (C20-D3-vitamin A), was designed to reduce the rate at which retinaldehyde reacts, reducing dimerisation and thereby potentially decreasing the accumulation of toxic byproducts.^42^ Administration of C20-D3-vitamin A in rodents with no genetic defects in vitamin A processing, decreased the rate of A2E biosynthesis.^43^ A randomised, placebo-controlled phase III study (SAGA study) of oral ALK-001(C20-D3-vitamin A) in subjects with GA due to AMD (n=300; NCT03845582) has commenced; with an estimated primary completion date of December 2021.

### Anti-inflammatory agents and complement inhibitors

**Figure 1:**
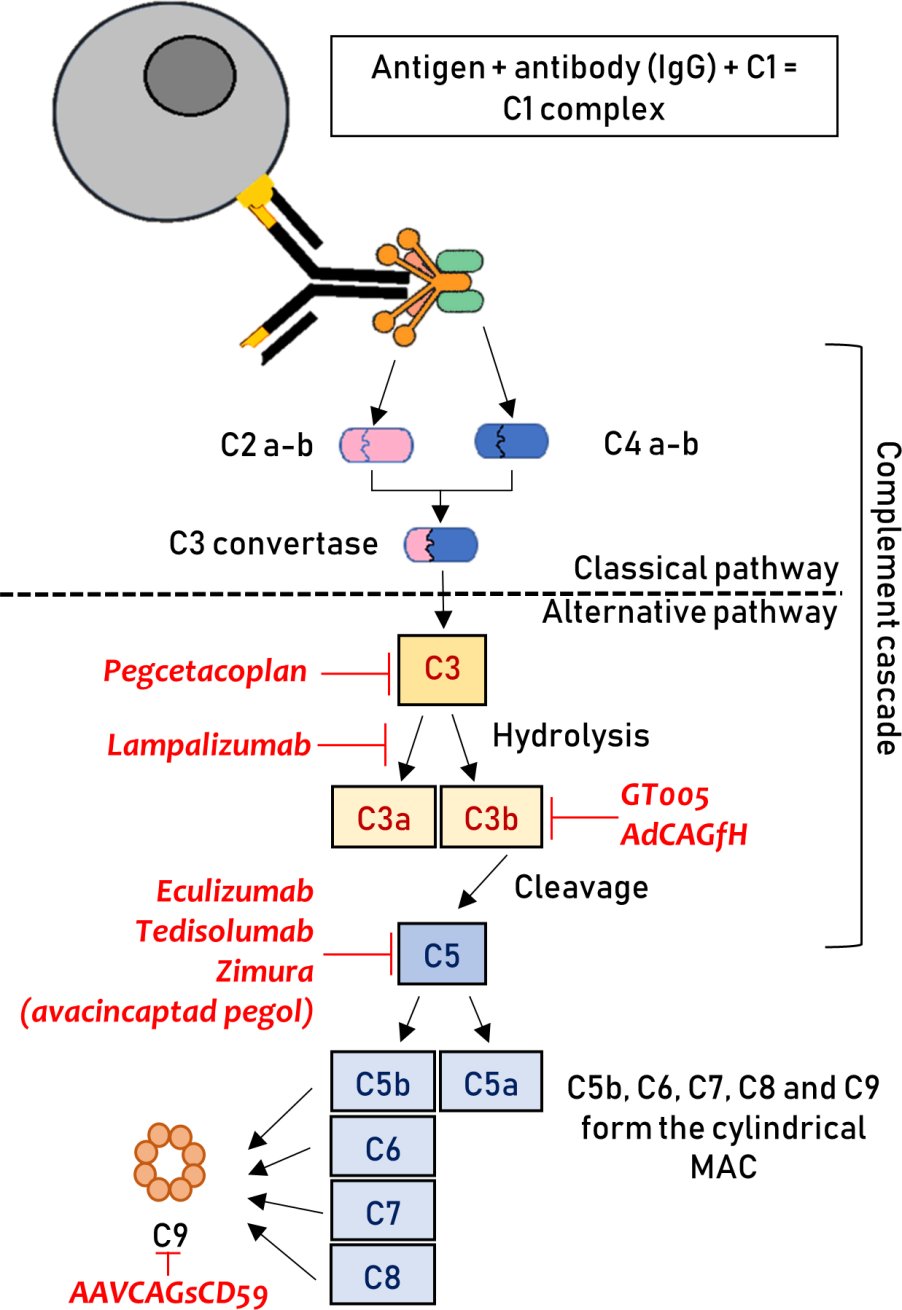
Graphical representation of the complement cascade. The location where different therapeutics act is highlighted. The therapies are shown, with them all have a downregulating effect on the particular step, negatively impacting on the downstream pathway. This figure has been created by the authors. C, complement; IgG, immunoglobulin G; MAC, membrane attack complex.

Several treatment strategies that modulate the complement system in patients with AMD are currently being investigated (figure 1).

#### Eculizumab

Eculizumab is an IgG antibody involved in the inhibition of complement component 5 (C5) that has been FDA-approved for the treatment of atypical haemolytic uremic syndrome and paroxysmal nocturnal haemoglobinuria, both resulting from complement cascade dysregulation.^44 45^ The COMPLETE study (n=30; NCT00935883) was a prospective, double-masked, randomised phase II clinical trial that evaluated the safety and efficacy of intravenous eculizumab for reducing the growth of GA in AMD.^46^ No drug-related adverse events were identified and none of the eyes converted to nAMD during a follow-up of 52 weeks. The drug did not decrease the growth rate of GA significantly; GA enlarged by a mean of 0.37 ± 0.22 mm in the eculizumab-treated eyes and by a mean of 0.37 ± 0.21 mm in the placebo group (p=0.93, 2 sample t-test).^46^

#### Lampalizumab

Lampalizumab is an antigen-binding fragment of a humanised monoclonal antibody that inhibits complement factor D.^47 48^ This factor has a key role in the alternative complement pathway, enabling the formation of C3b or C5 convertase. The CHROMA (n=906; NCT02247479) and SPECTRI (n=975; NCT02247531) trials were two identically designed, phase III, randomised controlled trials that administered 10 mg of intravitreal lampalizumab and the largest GA trials conducted to date. Although it was deemed safe, it did not reduce GA enlargement as compared with the sham group during the 48 weeks of follow-up.^49^

#### Sirolimus

Sirolimus (rapamycin) is a mammalian target of rapamycin inhibitor and immunosuppressive agent that is used for preventing organ rejection.^50^ It was administered subconjunctivally in a phase I/II trial (n=11; NCT00766649) in eyes of patients with GA. Subjects received 440 µg of subconjunctival sirolimus every 3 months. The investigational agent was well tolerated in all patients, but no positive anatomical or functional effects were identified and the rate of GA area growth did not decrease in the treated eyes as compared with the contralateral eyes.^51^

#### Avacincaptad pegol

Zimura (avacincaptad pegol) is an anti-C5 aptamer designed to decrease the activation of inflammasomes and the formation of MAC. A phase II/III, randomised controlled trial (n=286; NCT02686658; GATHER1) assessed the safety and efficacy of intravitreal administration of Zimura in subjects with GA. Published data showed that the investigational product met its prespecified primary endpoint—defined as reduction in the rate of GA growth measured by FAF—and was granted fast-track designation by the FDA.^52^ Over a follow-up of 12 months, patients treated with a 2 mg and 4 mg dose achieved a 27.3% (p value=0.0072) and 27.8% reduction (p value=0.0051), respectively, in the mean rate of GA growth as compared with the sham group.^53^ CNV was reported in the fellow eyes of 10 participants (3.5%), in the study eye of 3 participants (2.7%) in the sham cohort, 1 participant (4%) in the avacincaptad pegol 1 mg cohort, 6 participants (9%) in the 2 mg cohort and in 8 participants (9.6%) in the 4 mg cohort. Eighteen-month follow-up data are in keeping with aforementioned 12-month data, with a reduction in the mean rate of GA progression of 28.1% in the 2 mg group and 29.9% in the 4 mg group.^54^ Avacincaptad pegol was generally well tolerated and the adverse events were deemed to be related to the injection procedure.^52^ A second confirmatory phase III trial is underway (GATHER2).

#### Pegcetacoplan

Pegcetacoplan (APL-2) inhibits the complement component 3 (C3). FILLY was a phase II, multicentre, placebo-controlled, randomised trial (n=246; NCT02503332) that studied the safety and efficacy of intravitreal APL-2 in patients with GA. Patients receiving pegcetacoplan monthly or every other month (EOM), showed a GA growth rate reduction of 29% (p=0.008) and 20% (p=0.067), respectively, as compared with the sham treatment group,^55^ although no differences were observed in BCVA outcomes between the groups. Adverse events included an increased incidence of exudation in treated eyes of 8.9% in the EOM group and 20.9% in the monthly group. Two patients in the monthly group and one subject in EOM group developed endophthalmitis. The FDA granted pegcetacoplan fast-track designation, and enrolment for the DERBY (NCT03525600) and OAKS (NCT0355613) phase III studies are now completed.^56^

#### Tedisolumab

A phase II, randomised trial (n=150; NCT01527500) administered intravitreal LFG316 (tesidolumab), a complement component 5 (C5) inhibitor, in patients with GA. After 12 months of follow-up, there was no statistically significant difference in GA area—the primary endpoint—between the treated and sham groups. There were no serious adverse events related to the study drug.^57^

#### Risuteganib

Risuteganib is an intravitreally administered integrin inhibitor, which binds and inhibits certain types of integrin heterodimers that are thought to be involved in the development of AMD.^58^ In a phase II, placebo-controlled study, 1 mg of risuteganib was administered in patients with intermediate non-exudative AMD (NCT03626636; n=42). The primary endpoint was defined as the percentage of the population with ≥8 letters ETDRS BCVA gain from baseline to week 28 versus from baseline to week 12 in the sham arm. The results showed that the primary endpoint was met in 48% of patients in the risuteganib group compared with 7.1% in the sham group, which was statistically significant. No serious adverse events were reported.^59^

### Neuroprotective agents

Neuroprotection is another avenue with positive results in preclinical studies. If viable, it could lead to reduction of apoptosis and eventually halt the progression of GA in AMD. These drugs include antiapoptotic agents like tauroursodeoxycholic acid (TUDCA),^60–62^ dopamine-related therapies^63^ and growth factors such as ciliary neurotrophic factor (CNTF),^64^ among others.^65^

#### Ciliary neurotrophic factor (CNTF)

Evidence from preclinical studies encouraged a phase I trial (n=10; NCT00063765) to evaluate the safety of CNTF delivered over a 6-month period by encapsulated cells implanted intravitreally. The drug was deemed safe, with side effects being limited to low-grade anterior chamber activity that did not require treatment and a surgery-related choroidal detachment that resolved after the administration of topical steroids.^66^ Similarly, a placebo-controlled phase II study (n=51; NCT00447954), administered CNTF with the encapsulated cell technology intravitreally. The treatment group showed no difference in GA area or improvement in BCVA.^67^

#### Brimonidine tartrate

Brimonidine is an alpha2-adrenergic receptor agonist that has been established as an intraocular pressure-lowering agent, but promising evidence from clinical trials also suggests it has neuroprotective properties.^68 69^ It has been shown previously to promote survival and reduce photoreceptor damage in several animal models of acute retinal ischaemia,^70 71^ partial optic nerve crush,^72^ chronic ocular hypertension^73^ and in a light-induced model of progressive GA.^74^

The Brimonidine Drug Delivery System (Brimo DDS) is a sustained-release intravitreal implant. It was used in a randomised, placebo-controlled phase II trial (n=113; NCT00658619), in which study eyes were treated either with 132 µg, 264 µg or sham procedure. GA area growth at month 12 was 1.78 mm^2^, 1.59 mm^2^ and 2.19 mm^2^ in each group, respectively, which was statistically significant (p≤0.032). Adverse events were deemed as mild and related to the injection procedure.^75^ Subsequently, a larger placebo-controlled phase IIb study (BEACON study; n=303; NCT02087085) used a new generation device with a higher dose (400 µg) administered every 3 months in patients with GA. At 24 and 30 months of follow-up, GA area growth was reduced by 10% and 12%, respectively, in the treatment group. The study was terminated early due to a slow GA progression rate of ~1.6 mm^2^/year in the enroled population. However, the drug significantly reduced GA growth at the 30 months analysis, with mild adverse events which were transient and limited to the injection site^76^; thereby resulting in an ongoing phase III trial.^77^

### Gene therapy

Another treatment modality being explored is gene therapy. Due to the multifactorial and complex nature of AMD, there are multiple therapeutic challenges. Gene therapy interventions in AMD focus on the sustain expression of antiangiogenic and anticomplement proteins.

#### Targeting MAC formation

Two active phase I trials are using intravitreal AAVCAGsCD59 for both dry AMD and nAMD (NCT03144999 and NCT03585556, respectively). This compound acts as a membrane-bound inhibitor that reduces MAC formation (figure 1).^78^ It was previously shown to be successful in the attenuation of laser-induced CNV in mouse models, in both subretinal and intravitreal modes of delivery.^79^ Interim results of NCT03144999 (n=17) demonstrate the treatment to be well tolerated, with no dose-limiting toxicity. Four (23.5%) eyes developed mild inflammation that resolved with topical steroids or observation, and two of these four subjects also required topical medication to treat raised intraocular pressure.^80^

A different MAC approach is being evaluated in an ongoing phase I/II trial to study the safety and efficacy of subretinal GT005 (n=35; NCT03846193). GT005 was also designed to regulate complement activation and the formation of MAC (figure 1). It consists of a recombinant non-replicating adeno-associated viral (AAV) vector encoding complement factor I (a down-regulator of the C3b breakdown cycle), as a means to downregulate the alternative pathway.^81^ Subretinal administration of GT005 has been shown to significantly impact complement-driven CNV in a mouse laser-induced CNV model.^82^ The study is currently ongoing and has an estimated primary completion date of June 2021.

#### Complement factor H

Subretinal AAV-mediated expression of complement factor H (AdCAGfH, OMIM #134370) has been shown to attenuate C3-induced pathology in murine models, which is now under development for gene therapy in human subjects.^83^

### Cell-based therapies

#### Palucorcel

Palucorcel (CNTO-2476) is a human umbilical cord tissue-derived cell compound that has been previously shown to reduce functional deterioration in a rat model of retinal disease.^84^ A phase I/II dose-escalation study (n=35; NCT01226628), where the subretinal administration of CNTO-2476 via an ab externo surgical approach using the iTrack Model 275 microcatheter was evaluated in patients with GA. There was a high rate of adverse events related to the delivery procedure—17.1% (6/35) of subjects experienced retinal detachments and 37.1% (13/35) experienced retinal perforations—although, after 1 year, 34.5% (10/29) and 24.1% (7/29) experienced a ≥10 and ≥15-letter gain in BCVA, respectively.^85^

#### RPE transplantation

Two separate phase I/II trials have evaluated safety of RPE transplantation in patients with advanced dry AMD-related GA (NCT01344993) and Stargardt disease (NCT01345006). In the AMD trial (n=9), RPE cells derived from human embryonic stem cells (MA09-hRPE) were delivered to the subretinal space after standard vitrectomy. There was no evidence of adverse proliferation, rejection or serious ocular or systemic safety concerns, with the adverse events deemed to be associated to the surgery and immunosuppression. Six (66%) out of nine patients showed a BCVA improvement of at least 11 ETDRS letters and the other three subjects remained stable (defined as change of ≤10 letters).^86^ Combined results of the two trials showed that out of the 18 patients enroled with either Stargardt disease or AMD-related GA, 72% (13/18) had patches of increased subretinal pigmentation at the border of the atrophic areas.

Another phase I/IIa trial (n=16; NCT02590692), administered a polarised monolayer of human embryonic stem cell-derived RPE cells (CPCB-RPE1) on a parylene membrane in the subretinal space of subjects with GA. Interim data revealed that the product was successfully implanted in four subjects, with an improvement of 17 ETDRS letters in one patient. None of the implanted eyes showed progression of vision loss after 1 year of follow-up.^87^

### Mitochondrial enhancers

Elamipretide is a cardiolipin-protective compound that protects the structure of the mitochondrial cristae and promotes oxidative phosphorylation.^88^ In a mouse model of sub-RPE deposits (ApoE4 transgenic mice fed high fat and cholesterol), mitochondrial dysfunction was suggested to be a trigger of deposit formation, which was successfully reversed with subcutaneous elamipretide.^89^ The ReCLAIM study was a phase I trial (n=40; NCT02848313), designed to evaluate the safety and tolerability of subcutaneous elamipretide in patients with intermediate AMD (including a high-risk drusen without GA subgroup and a non-central GA subgroup). The outcomes were assessed at week 24. Patients with non-central GA (n=15) showed a mean increase in low-luminance visual acuity of 5.4 ± 7.9 letters and BCVA of 4.6 ± 5.1 letters. The patients with high-risk drusen (n=19) also demonstrated improvements in low luminance and BCVA. Adverse events were mostly limited to reactions in the injection site.^90^ A placebo-controlled phase II trial, ReCLAIM-2 (n=180; NCT03891875) is currently enrolling.

### Nanosecond laser therapy

The capacity of subthreshold nanosecond laser treatment to reduce drusen and reduce Bruch’s membrane thickness, while maintaining retinal structure, was assessed in the Laser intervention in early stages of AMD (LEAD; n=292; NCT01790802) randomised trial, after promising results in preclinical studies.^91^ Overall, there was no significant difference in the progression rate to late AMD between those receiving nanosecond laser and the sham group. A post hoc analysis showed evidence of effect modification based on the coexistence of reticular pseudodrusen (RPD), where progression was slowed down after the laser treatment in participants without coexistent RPD at baseline and accelerated in those with RPD.^92^

## Conclusions

AMD is a complex disease with several pathways plausibly involved in its pathogenesis, which poses therapeutic challenges. For the time being, the management of dry AMD depends greatly on observation, lifestyle changes, frequent follow-up evaluations, early recognition of visual deterioration and CNV detection. Several therapeutic avenues to reduce the rate of disease progression are being investigated, including (1) drugs with antioxidative properties, (2) inhibitors of the complement cascade, (3) neuroprotective agents, (4) visual cycle inhibitors, (5) gene therapy and (6) cell-based therapies, among others.

Several early phase clinical trials have shown significant promise, creating high expectations from the associated expansion phases that are ongoing or anticipated in the near future. In contrast, several trials have failed to meet primary and secondary endpoints, despite promising results in animal models and ex vivo. The main difficulties have revolved around the various methods of drug delivery, the lack of efficacy in reducing the rate of atrophy progression as compared with control eyes, and safety challenges. It is still unclear if some of the investigational agents used to treat dry AMD can predispose or accelerate the rate of progression to nAMD.

Nevertheless, given the enormous and increasing burden of disease that dry AMD represents, continued rapid development is expected, with the number of clinical trials increasing into the foreseeable future—and optimism that a meaningful treatment will be approved in the near future.
